# Development and validation of risk factors lifestyle disorders scale (RFLDS): A cohort study

**DOI:** 10.1016/j.dialog.2026.100307

**Published:** 2026-05-03

**Authors:** Kalyani Singh, Ritu Pradhan

**Affiliations:** Department of Foods and Nutrition, Government Home Science College, Chandigarh, India

**Keywords:** Lifestyle disorders, Scale, Adolescents, Health, Lifestyle, WHO, Sustainable development goal 3.4

## Abstract

**Background:**

Adolescents are presumed to be healthy, but being a vulnerable group they are threatened with morbidities. Management of risk factors which concern and affect lifestyle disorders must begin during adolescence. The aim of study is to develop and validate a scale to assess risk factors leading to lifestyle disorders in quick and concise manner.

**Methods:**

Risk Factor Lifestyle Disorders Scale (RFLDS) has been developed to identify risk factors of lifestyle disorders on low, borderline and high risk zones based on scientific exhaustive review of literature and expert consensus. Cross-sectional baseline data from planned cohort of 1000 school-based adolescents aged between 16 and 18 years from Tricity- Chandigarh, Panchkula and Mohali (India) was carried out. Three phase validation of content, construct and criterion validity has been conducted to develop the scale. Analyses were done through Item Total Reliability, Exploratory Factor Analysis, Cronbach's Alpha and Pearsons Correlation.

**Findings:**

Results layout a structure of developed and validated scale. KMO measure of 0.720, Bartlett test of *p* < 0.0001, leading to seven factors consisting of 19 items of 64.75% variance and Cronbach's alpha of 0.68 was observed. Final scale consists of seven factors that emerged out of 19 identified items namely Socio-Economic Criteria (SEC), Nutritional Status (NS), Body Composition (BC), Low Risk Factor Foods (LRF), High Risk Factor Foods (HRF), Mental Health and Family and Personal History (MHFPH) and Sleep and Physical Activity (SPA).

**Interpretation:**

Lifestyle disorders lead to deterioration in quality of life. Comprehensive *RFLDS- Risk Factors Lifestyle Disorders Scale,* validated in adolescents only, may be used for easy and quick assessment, addressal and prevention of lifestyle disorders. It may also be used as a screening tool in institutes, clinical and public health care systems to improve overall well-being, aligning with WHO's Sustainable Development Goal 3.4.

## 1 Introduction

Adolescents account for 1.2 billion or 18% of the global population [1]. The developing countries encompass 88% adolescents wherein India accommodates 253 million, constituting to every fifth individual being an adolescent [2]. Adolescence, is a noteworthy period and must be dealt well with proper nutrition and relevant information for good health and apt development of self [3]. Lifestyle disorders, also known as Non-Communicable Diseases (NCD) and lifestyle diseases include hypertension, diabetes stroke, cancer, heart diseases as well as chronic lung diseases [4]. Initially known as ‘Diseases of Affluence’, today they are internationally known as NCDs under the umbrella of chronic degenerative diseases [5]. The non-communicable diseases accounted for 71% deaths in 2016 [6] which have increased to 74% death as per the latest statistics [4].

Adolescence being a vulnerable phase and an important juncture of life, can be impacted both negatively and positively by various lifestyle changes. This period must be dealt in such a way that it carves a road map towards holistic health [1], [7]. With the current global scenario, it is pertinent to grasp and comprehend challenges and strategize ways towards having adolescents with a healthy lifestyle [8]. According to World Health Organization [4], the three major risk factors for lifestyle diseases are lack of physical activity, unhealthy diet and smoking. The NCDs are also a potential threat towards attaining Sustainable Development Goals. Reduction of Non-Communicable Diseases is one of the goals in the Sustainable Development Goals (SDGs): 2030 Agenda. The aim of SDG Target 3.4 is to fortify the universal health coverage for a comprehensive health care and well-being of individuals [8].

Referring to the developing country which houses maximum adolescents i.e. India, the 2016 ICMR State Level Disease Burden Initiative data showcases that as many as six million deaths have been caused due to one or another lifestyle disease [9]. As per the National Family Health Survey (NFHS)-5 [10] data, there has been an increase in women population (aged 15–49 years) with BMI >25 kg/m^2^ i.e. 24% against 20.6% of NFHS 4 [10] data. Similar trend has been observed among men population (aged 15–49 years) wherein 22.9% have been noted with BMI >25 kg/m^2^ against 18.9% of NFHS 4 [10] data. A drift towards overweight and obesity has been observed among the population. This could be due to a transition in the everyday life patterns among both women and men of India, attributing to lifestyle diseases.

The predominant risk factors of these lifestyle diseases include less physical activity, faulty dietary habits, excessive intake of tobacco and alcohol. The basic metabolic risk factors are hypertension, BMI >25 kg/m^2^, hyperlipidemia and hyperglycemia [8]. Incorrect eating habits, sedentary lifestyle and excessive screen time are cogent factors that have increased the risk of lifestyle disorders [11], [12]. Further, ICMR-NIN Expert Committee [13] has highlighted that the three main ingredients in ultra processed foods are added sugar, fats and salt which increase the risk of lifestyle disorders. Prasad et al. [14] also deduced that blood pressure is also a risk factor of lifestyle disorders and it is important to keep a tab on blood pressure readings as it is not checked on a regular basis among adolescents. Thomas et al. [15] have discussed TOFI- thin on the outside, fat on the inside. Two individuals who have similar BMI levels can have varied body fat percentage in them representing their lifestyle. It is therefore essential that to take note of high body fat percentage as a risk factor of lifestyle disorders. Body water percentage is inversely proportional to body fat percentage, waist circumference and body weight [16]. Family history also facilitates the prediction of some lifestyle diseases [17].

Overall, transformation to a healthy life pattern is the key to healthy society and nation as a whole [18].

Keeping an optimistic approach along with low BMI levels, exercise, nutritious balanced diet, optimistic attitude, no substance abuse such as smoking and alcohol consumption and social rhythm may reduce the risk of chronic degenerative diseases. This will aid in keeping health in fine fettle [19], [20]. With respect to dietary parameters, which is a major contributor to lifestyle disorders, Schulze et al. [21] have comprehended the association between eating habits and lifestyle diseases. Higher consumption of whole grains and legumes, fruits and vegetables, fish, nuts and fermented dairy products aid in preventing lifestyle diseases. Indian Council of Medical Research- National Institute of Nutrition [22] also stresses on the same.

### 1.1 Existing measures

Adolescence is a phase which brings about various opportunities for healthy lifestyle paving way for a healthy future. It is imperative to regularly evaluate risk factors of lifestyle diseases among adolescents, a vulnerable and critical segment of our population to prevent them from suffering later in life. Constant and systematic risk assessment along with apt interventions are necessary to prevent lifestyle disorders among them to maintain their health status [23]. As per latest literature, innovative models are needed for screening of risk factors of lifestyle disease and developing a tool for assessment [24]. Lifestyle diseases can be comprehended through the Health Belief Model which conceptualizes screening and perception of health threats and taking decisions towards improvement for achieving healthy lifestyle goals. The framework of the Model assists to perceive susceptibility and severity by assessing various risk factors of lifestyle diseases [25].

Adolescent Healthy Lifestyle Questionnaire (AHLQ) developed by Taymoori et al. [26] measures healthy lifestyle among adolescents through 36 items under six categories- life appreciation, health responsibility, nutrition, social support, physical activity and stress management. Cronbach's alpha measuring reliability was 0.82. Though AHLQ promotes changes for a healthy lifestyle among adolescents, but the scale does not delve into the anthropometric parameter, which is quite essential for assessing risk of lifestyle diseases. It does not explore some other pertinent risk factors encompassing clinical profile i.e. blood pressure, sleep time and family history of lifestyle diseases. The aim of the current tool is to incorporate these factors in the present scale.

Keeping Indian adolescents in purview, Long et al. [27] developed and validated Indian Adolescent Health Questionnaire (IAHQ). This includes 12 modules- demography, physical health, physical activity, nutrition, hygiene, medical care, tobacco, alcohol and drugs, domestic violence and violence, injury and mental health, family, home and school environment. The scale covers quite some risk factors of lifestyle diseases and every parameter has its own likert scale of scoring. Cronbach's alpha was 0.65 between substance abuse and mental health modules and of 0.40 between physical activity, hygiene, nutrition, safety, exposure to violence and relationship with parents. The IAHQ covers majority of the lifestyle diseases risk factors, but every risk factor has its own style of scoring. There is no convergence of these risk factors into uniform scoring to make it easier for an individual to assess his or her risk factors for lifestyle diseases. This limitation has also been worked upon in the current scale by carrying out composite scoring for uniformity in every item by putting it under low, borderline and high risk zones so that it is less complicated for the adolescents. Reis et al. [24] developed a toolkit on healthy lifestyle assessment with the intent to inculcate knowledge and improve the quality of life among people. The toolkit consists of eight parameters- anthropometry, physical activity, social well-being, nutrition, mental health, use of substance as well as smoking and drinking, sleep, and health and disease. The questions were rated on the basis of traffic lights system of green light showing low risk, yellow light meaning moderate risk and orange light depicting high risk making it easy for the people to maintain their lifestyle. After thorough review of literature, it was deduced that there is no such scale which incorporates and assesses all the risk factors such as BMI levels, waist circumference, body composition, blood pressure, sleep time and family history of any disease in the most uniform and compact way with composite scoring method. The participant should just fill in required information and perceive where his or her health stands.

Since adolescents fall in a vulnerable and critical section of population in India, correct lifestyle and behavioural choices can pave way to a healthy lifestyle. With Non-Communicable Diseases being on the rise due to defective lifestyle, identification of its risk factors during adolescence itself is pertinent so that they can be addressed and prevented in an early phase.

Thus, the present study aims to fill the gaps in AHLQ and IAHQ through a newly formed measure- Risk Factor Lifestyle Disorders Scale (RFLDS). The current validated RFLDS is thus, a 19-item, seven-factor Scale which is inclusive of anthropometric measurements, blood pressure, sleep time and family history of lifestyle diseases along with a composite scoring method. The scale has been developed by rigorous evaluation of its content, construct and criterion validity to include, confirm and verify all relevant aspects of the concept. The KMO measure of 0.720, Bartlett test of *p* < 0.0001, variance of 64.75% and Cronbach's alpha of 0.68 has been observed. The current scale aims for quick and easy identification of risk factors of lifestyle disorders for addressal, prevention and screening. It has been developed to educate and improve the health status as well as foster quality of life of adolescents.

## 2 Methods

This manuscript reports cross-sectional baseline findings from a planned cohort study. A quantitative approach was adopted in the current study. Multistage stratified random sampling method was carried out to obtain data from adolescents in Tricity. The language of the scale is English.

### 2.1 Sample

**Fig. 1:**
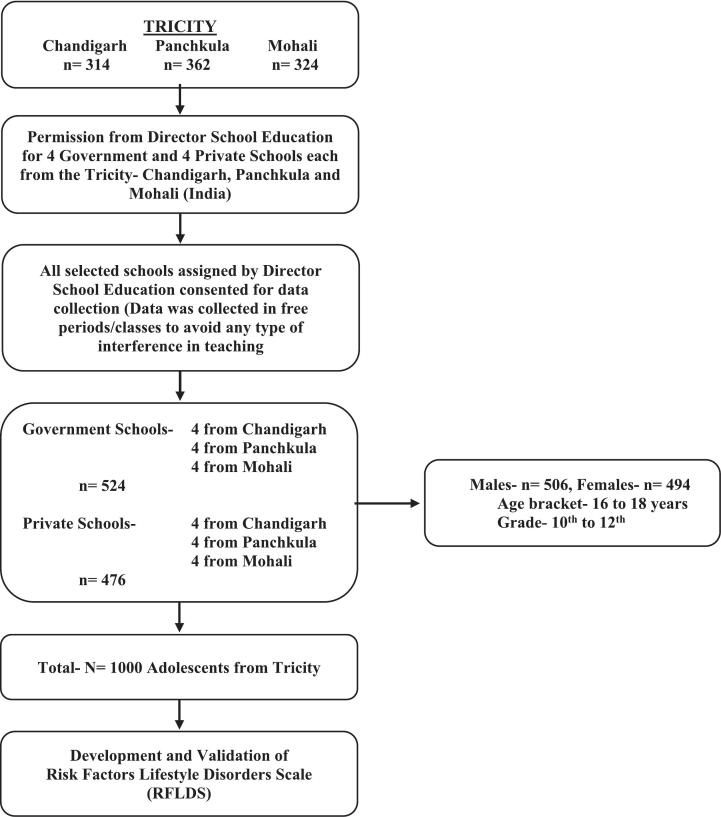
Flow figure of participants/ adolescents.

Keeping in mind the surge of Non-Communicable Diseases and the critical section of India's population i.e. adolescents, the current cohort study was carried out to evaluate the incidence of low, borderline and high risk zones of various risk factors of lifestyle disorders among 1000 adolescents/participants. Through the multistage stratified random sampling, the population was stratified to three stages- area, schools and gender. This integration aided towards approaching and taking an appropriate sample size for the research. Firstly, the Tricity i.e. Chandigarh, Panchkula and Mohali (India) was selected on the basis of NFHS-5 [10] data. The schools taken here are Government and Private schools from where male and female participants between 16 and 18 years of age from 10th to 12th grades were randomly selected. The data thus comprised of 494 female and 506 male participants studying from Government (*n* = 524) and Private (*n* = 476) schools from Chandigarh (*n* = 314), Panchkula (*n* = 362) and Mohali (*n* = 324), as seen in Fig. 1. The observations were treated independently. No adjustments were made for clustering with respect to schools, which might narrow some confidence intervals. Chandigarh, known for its 20th century modern architecture and urban planning is located in the foothills of Himalayas, and capital city of Punjab and Haryana. Panchkula and Mohali are planned districts of Haryana and Punjab respectively [28]. 74.4% participants belonged to urban areas and 25.6% belonged to rural areas.

### 2.2 Procedure

Permissions to collect data for the current research was taken from the Department of Education of Chandigarh, Panchkula and Mohali. Necessary permissions from respective schools' Principals were also taken to fix dates for data collection. Class incharges were met to fix the timings so that the studies of participants may not be affected. The test was administered by giving a brief introduction of the purpose of the research. The questionnaire was partly administered on the participants to collect data for body mass index, blood pressure, waist circumference, body fat percentage and total body water percentage items. The rest of the items were self-administered. The current study involved anonymous, minimal risk surveys as there were no invasion, pain injury or radiation. Principle of professional competence was followed whereby the research was planned, conducted, evaluated and monitored throughout at all stages by persons who were competent and have the appropriate and relevant technical and requisite qualification, experience and training. These were conducted within school premises, overseen by teachers in charge who reported to the Principals of the respective schools under the Department of School Education. Ethical guidelines laid down by the Indian Council of Medical Research [29] were followed. The participation was carried out after their voluntary consent; confidentiality of data was maintained; no blood sample was taken; and well-being of society has been prioritized during this research [29].

### 2.3 Data analysis

The following statistical analyses were done using Statistical Package for Social Science (SPSS) version 26.

### 2.4 Item analysis

The questionnaire was then filled in by 100 students which underwent item analysis through item total reliability to apprehend the indicators for validity.

### 2.5 Construct validity

The scale was administered on the sample of 1000 participants for Exploratory Factor Analysis (EFA) using principal component analysis. Exploratory Factor Analysis (EFA) was run on data of 1000 participants using principal component analysis through orthogonal (varimax) rotation. Through this analysis, the items got grouped into factors which were noted.

### 2.6 Criterion validity

To check the scale's validity, 10% i.e. 100 same participants were also requested to fill the Indian Adolescent Health Questionnaire of Long et al. [27]

### 2.7 Development of RFLDS

• Item Generation and Identification of Risk Factors

Generation of potential items qua various risk factors of lifestyle disorders were generated by exhaustive scanning of literature review. Key words such as lifestyle disorders with nutrition, anthropometry, socio economic status, gender, body composition, physical activity, substance, sleep, screen time, stress, history of any lifestyle disease etc. were used to avail information.

**Fig. 2:**
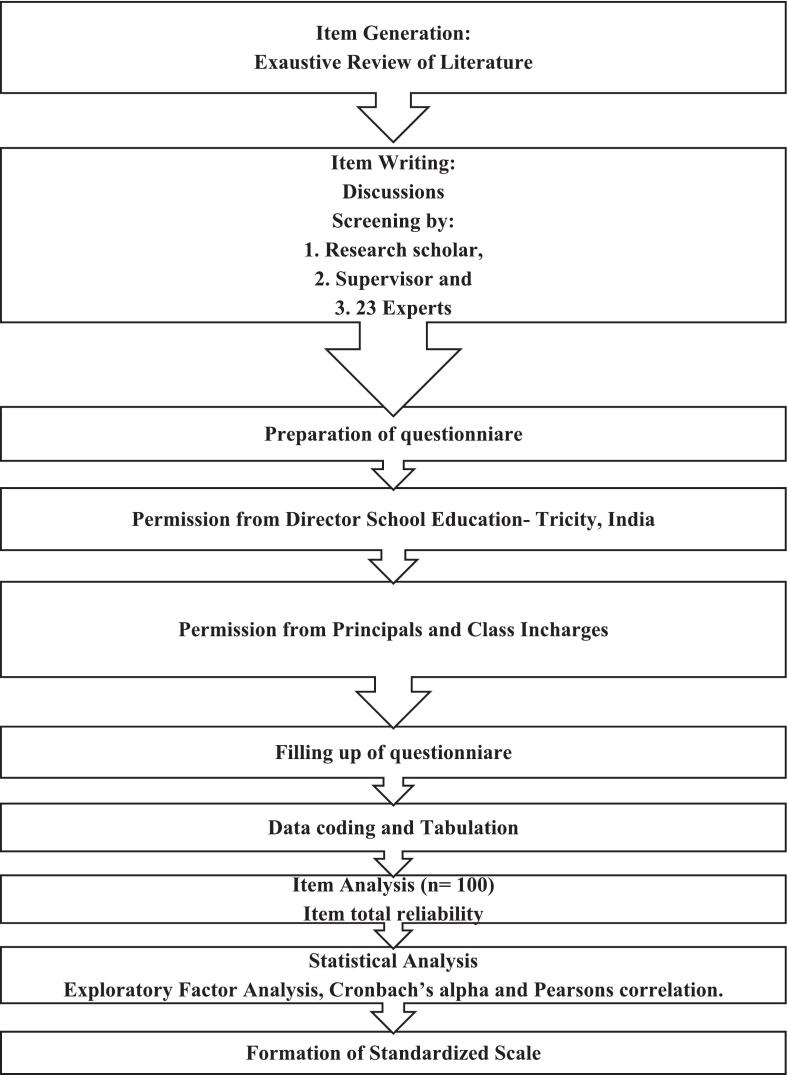
Flowchart of development of standardized scale.

With this, the initial pool of items was drafted into a questionnaire. The questions were kept simple, short, crisp and unambiguous. The scoring of risk factor items of lifestyle disorders has been bifurcated on the basis of low, borderline and high-risk factors on the basis of thorough review of literature from authentic sources and expert consensus. The developed current scale has been validated under three sub-heads: content validity, construct validity and criterion validity as seen in Fig. 2.

• Content Validity

The questions under various risk factor items were further scrutinized and analysed. The questionnaire was first screened by the research scholar followed by the supervisor. The repetitive and redundant questions were removed and the items which were superfluous, vague and ambiguous were eliminated. This was sent for content validation to 23 experts and academicians from the fields of food and nutrition, endocrinology, gynaecology, physiology, physical education, psychology, sociology and commerce. A four-point likert scale with scoring ranging from least relevant- 01 to highly relevant- 04 [30] was used for evaluation. Feedback, suggestions and criticism if any was requested to be provided in a textbox under each item. Content Validity Index was evaluated as per the scoring done by experts [31]. Both Item level (I-CVI) and Scale level (S-CVI) CVI were assessed. The I-CVI assessment was taken from 0 to 1. The I-CVI score above 0.79 was treated as relevant, between 0.70 and 0.79 needed revision and below 0.70 were to be eliminated [32]. No item got deleted but two items with score of 0.71 were revised for better understanding and clarity. Table 1 shows the Content Validity Index, I-CVI and S-CVI/Ave. A total of 21 items were generated to assess the risk factors of lifestyle disorders that were put in a uniform way, with composite scoring to tell low, borderline and high-risk zones.

**Table 1:** Content validity index.

Expert No.	I-CVI	I-CVI interpretation
1	0.81	Relevant
2	1	Relevant
3	1	Relevant
4	1	Relevant
5	1	Relevant
6	0.95	Relevant
7	1	Relevant
8	0.95	Relevant
9	0.95	Relevant
10	1	Relevant
11	1	Relevant
12	0.71	Needs revision
13	1	Relevant
14	1	Relevant
15	1	Relevant
16	0.90	Relevant
17	1	Relevant
18	1	Relevant
19	0.71	Needs revision
20	1	Relevant
21	1	Relevant
22	1	Relevant
23	1	Relevant
S-CVI/Ave	0.96	..

The initial pool of 21 items put together were:

1. Body Mass Index (BMI)
2. Blood Pressure (BP)
3. Waist circumference
4. Body fat percentage
5. Total body water percentage
6. Occupation of the Head of Family
7. Education of the Head of family
8. Family Income
9. Physical Activity
10. Screen Time
11. Sleep time
12. Substance abuse (smoking and alcohol consumption)
13. Intake of carbohydrates
14. Intake of refined and added sugar
15. Intake of fibre
16. Intake of fats
17. Intake of fruits and vegetables
18. Intake of sodium
19. Family history of lifestyle diseases
20. Personal history of lifestyle diseases
21. Mental health

## 3 Results

### 3.1 Item generation, content validity and item analysis

**Fig. 3:**
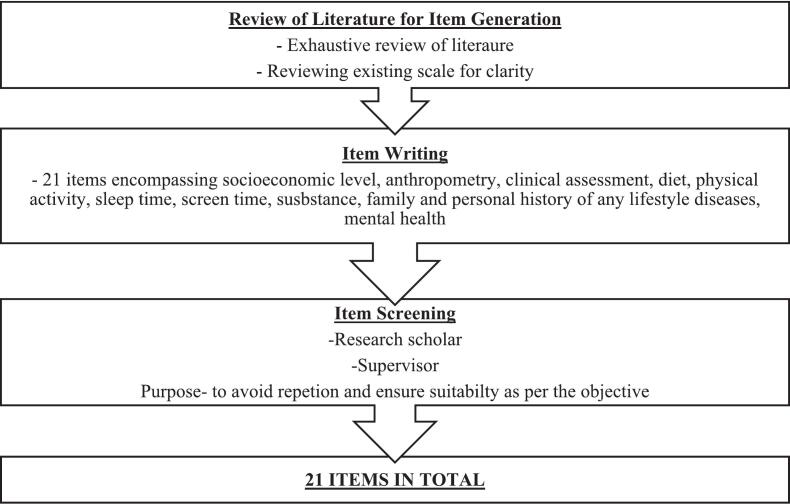
Flowchart of procedure of development of standardized scale - Item Generation.

**Fig. 4:**
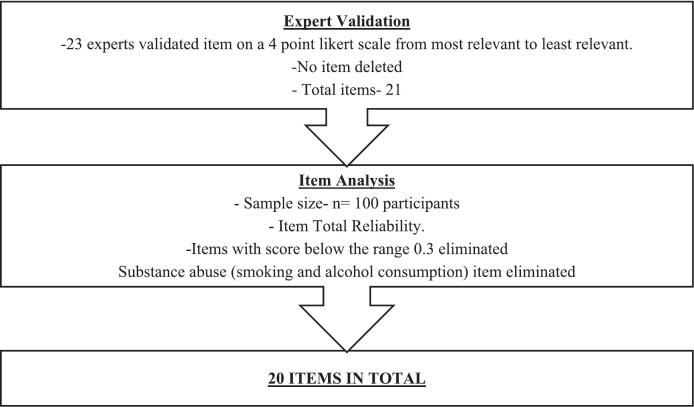
Content validity and item total reliability.

The potential 21 items in relation to lifestyle disorders were assembled together in the questionnaire. Some items in the scale such as BMI, BP, waist circumference, body fat percentage, total body water percentage, screen time, sleep time, occupation, education and family income, had direct answers to gauge low, borderline and high risk zones. Some questions were incorporated in the other items such as physical activity, substance abuse, nutritional intake, family and personal history of lifestyle diseases and mental health to assess risk factors. The scores of these items were then to be calculated, converging them into low, borderline and high risk zones for assessing possibility of lifestyle disorders. Thus, every item in the scale had converged uniformly into composite scoring, keeping the sanctity of relevance of every item, thus congregating them to put them on a similar pedestal among each other to bring out the scale [33]. As seen in Table 1, out of 21 items, the I-CVI score of 16 items was 1, three items was 0.95, one item was 0.90 and one item was 0.81. I-CVI score of two items was 0.71 which needed revision and were accordingly modified as per experts' suggestions. No item had I-CVI score below 0.70. Hence, all 21 items were retained in the questionnaire [31], [32]. The S-CVI/Ave score was 0.96 which met a satisfactory level of content validity [32]. The item analysis was then carried out on the basis of item total reliability. Those with score < 0.3 were deleted from the scale. This led to elimination of item ‘substance abuse’ which included smoking and alcohol consumption, leaving 20 items in the scale as seen in Fig. 3, Fig. 4.

### 3.2 Discussion of item generation, content validity and item analysis

The items and questions in the scale were double checked to avoid any repetition by the research scholar and supervisor.

Even though substance abuse is a primary risk factor of lifestyle disorders, but invalid answers possibly steered by social standing and consciousness of peers, led to its deletion. The valid responses might not have been received due to cultural sensitivity as smoking and consumption of alcohol is considered a taboo in India [34]. Hence, the participants might have refrained from answering in affirmative even if they were smoking or drinking alcohol. Also, the legal age of drinking in India is different in every State, overall being above 18 years [35].

### 3.3 Construct validity

**Fig. 5:**
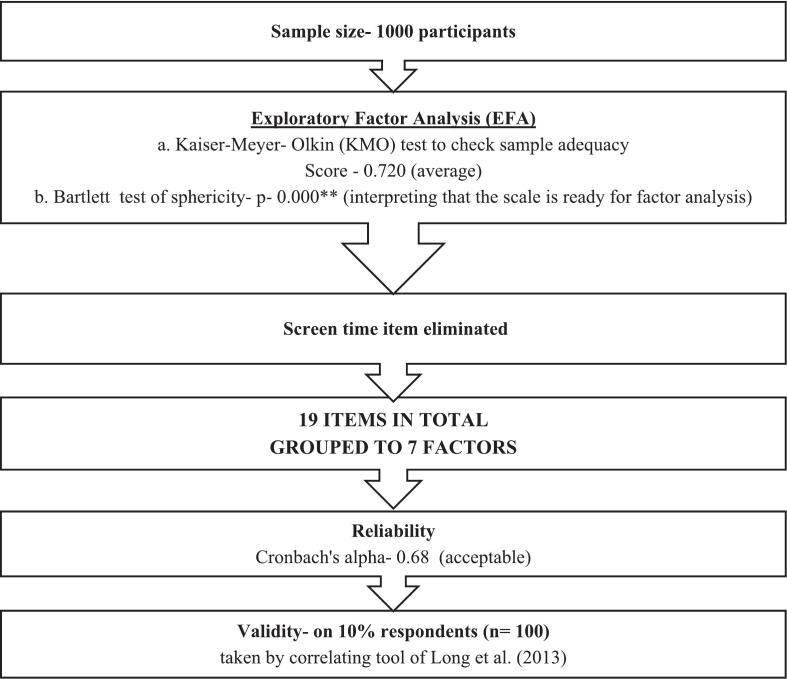
Construct and criterion validity.

Kaiser-Meyer-Olkin (KMO) measure of sample adequacy was 0.720 which is average [36]. Bartlett's test of sphericity showed the all-round relation between variables and total sample size to be highly significant (*p* > 0.0001**) to proceed towards factor analysis as seen in Table 2. Exploratory Factor Analysis (EFA) was run on data of 1000 participants. Principal component analysis through orthogonal (varimax) rotation was conducted to reduce the item pool and derive composite indices for further analysis instead of latent construct inference. Factors with eigenvalue >1 were extracted and considered. As a result, 19 items got regrouped together into seven factors. Factor loading of items >0.40 were extracted [37]. The items with cross loading >0.40 in more than a single factor were reviewed conceptually. Those with apt justification were retained. Cumulative variance of 64.75% was observed, which is acceptable [38] as seen in Table 3. Screen Time item value came out to be 0.376 and was eliminated. The values of the items with two decimal loadings have been shown in Table 3. Thus, the final scale comprised of 19 items as seen in Fig. 5.

**Table 2:** KMO and Bartlett's tests.

KMO and Bartlett's Test (n−1000)		
Kaiser-Meyer-Olkin Measure of Sampling Adequacy		0.720
Bartlett's Test of Sphericity	Chi-Square	5823.573
	df	190
	Sig.	0.0001⁎⁎

⁎⁎ *p* < 0.01.

**Table 3:** Item total reliability and exploratory factor analysis.

Factor	..	..	..	..	..	..	Component						
		Item Total Reliability	Eigenvalue	% of Variance	Cumulative %	Subscale Reliability	1 SEC	2 NS	3 BC	4 LRF	5 HRF	6 MHFPH	7 SPA
Nutritional Status (NS)	BMI	0.91	2.538	12.688	12.688	0.78	0.85	..	..	..	..	..	..
..	BP	0.83					0.86	..	..	..	..	..	..
..	Waist circumference	0.64					0.77	..	..	..	..	..	..
Socio-Economic Criteria (SEC)	Occupation	0.90	2.246	11.230	23.918	0.79	..	0.83	..	..	..	..	..
..	Education	0.95					..	0.83	..	..	..	..	..
..	Family income	0.75					..	0.81	..	..	..	..	..
Low Risk Factor Foods (LRF)	Fibre	0.87	2.064	10.322	34.240	0.72	..	..	0.84	..	..	..	..
..	Fruits and vegetables	0.89					..	..	0.75	..	..	..	..
..	Carbohydrates	0.58					..	..	0.73	..	..	..	..
Body Composition (BC)	Body fat percentage	0.86	1.844	9.218	43.459	0.81	..	..	..	0.89	..	..	..
..	Total body water percentage	0.70					..	..	..	0.88	..	..	..
High Risk Factor Foods (HRF)	Sugar	0.54	1.616	8.078	51.537	0.53	..	..	..	..	0.78	..	..
..	Sodium	0.82					..	..	..	..	0.68	..	..
..	Fats	0.63					..	..	..	..	0.65	..	..
Mental Health and Family and Personal History (MHFPH)	Mental Health	0.81	1.516	7.580	59.117	0.44	..	..	..	..	..	0.71	..
..	Family history of any disease	0.95					..	..	..	..	..	0.66	..
..	Personal history of any disease	0.98					..	..	..	..	..	0.65	..
Sleep and Physical Activity (SPA)	Sleep hours	0.88	1.127	5.634	64.751	0.58	..	..	..	..	..	..	0.854
..	Physical Activity	0.75					..	..	..	..	..	..	0.820

### 3.4 Discussion of construct validity

Table 3 shows 19-item Scale grouped into seven factors:

1. SEC (Socio-Economic Criteria)- Occupation, education and family income. It consists of three basic parameters- family income, education of the head of the family and the occupation of the head of the family. Since the socio economic criteria is a classified factor already, it has not been measured on the basis of low, borderline and high criterion.
2. NS (Nutritional Status)- BMI, BP and waist circumference. The values of these items will tell whether the individual falls under low, borderline or high risk zones.
3. BC (Body Composition)- Body fat percentage and total body water percentage. These two items are inversely proportional to each other. Thus, lower the value of body fat percentage, and higher the totally body water percentage, lower be the risk of lifestyle disorders.
4. LRF (Low Risk Factor Foods)- Fibre, fruits and vegetables and carbohydrates. As grouped by EFA, the intake of these items can be calculated through dietary recall. They are essential food sources that must be consumed in appropriate quantities everyday as per ICMR-NIN Expert Group [22]. The calculated values when put in the scale tell if the individual is under low, borderline or high risk zone.
5. HRF (High Risk Factor Foods)- Sugar, fats and salt intake. As grouped by EFA, these three items, common in ultra processed foods, can be calculated though dietary recall. High in calories and low in essential amino acids, fibre and micronutrients, these foods if consumed on a regular basis may increase the risk of lifestyle disorders. Some ultra processed foods commercially available are bakery items breakfast cereals, biscuits, chips, ice cream, tofu and its products, cheese, butter, frozen foods, plant based proteins and meats, power drinks, preserves such as jams and sauces and mayonnaise [13]. The calculated values when put on the scale will depict whether the individual is under low, borderline or high risk zone.
6. MHFPH (Mental Health and Family and Personal History)- These items, grouped together through EFA, are an imperial part of public health. Mental health has been dealt with parameters of stress, expectations and anxiety. Lower the score, better the mental health. For family and personal history, higher the number of lifestyle diseases in an individual, greater is the risk of further lifestyle diseases.
7. SPA (Sleep and Physical Activity)- The number of sleep hours and minutes of physical activity per day will aid to gauge whether the individual is in low, borderline or high risk zone.

The subscale reliability as measured on the basis of Cronbach's alpha shows Socio- Economic Criteria (SEC) factor as 0.79, Nutritional Status (NS) factor as 0.78, Body Composition (BC) factor as 0.81, Low Risk Factor Foods (LRF) as 0.72, High Risk Factor Foods (HRF) as 0.53, Mental Health and Family and Personal History (MHFPH) as 0.44 and Sleep and Physical Activity (SPA) as 0.58, as also seen in Table 3. Three factors, namely HRF, MHFPH and SPA had lower subscale reliability. This might be because the items in the respective factors measured diverse aspects of the construct. Hence, the internal consistency might have reduced to preserve the concept. The Cronbach's alpha depicting reliability was 0.68 revealing that the tool's acceptablity [39] due to it encompassing heterogenous constructs [40].

The final scale consists of 19 items with seven factors. Though the Cronbach's alpha is within acceptable range, it is however still necessary to continuously refine and test RFLDS. The confirmatory factor analysis will be conducted in future studies. The Risk Factor Lifestyle Disorders Scale, a sample of completed RFLDS, scoring sheet and brief user guide for practitioners has been attached.

For instance, RFLDS was filled by a 17-year-old male adolescent, as shown in the sample in supplementary material.

The factor Socio-Economic Criteria (SEC)- education, occupation and family income were noted. The highest qualification of the participant's head of family was 10th grade, then 10th in the education of head item was circled. The head of the family was the head clerk in a college, then skilled work was circled under the occupation of head item. The total family income per month was Rs.35,000/−, hence Rs. 25,001- Rs. 50,000/− per month was circled under the family income item. The score given for every circled item was added, which was 4 + 4 + 2 = 10. According to the score, the participant fell in the Upper Middle Income Group.

In the Nutritional Status (NS) factor, BMI, waist circumference and Blood Pressure items were assessed. The calculated BMI was 18.9 kg/m^2^. The 18.6–22.9 kg/m^2^ reading in the scale was circled which showed that the participant fell in the borderline risk zone, meaning thereby that the participant must take care of maintaining his BMI levels. The waist circumference was 82 cm or 0.82 m. Since the waist circumference of the participant was <0.95 m, it depicted low risk zone. Similarly, the Blood Pressure reading was 118/78 mmHg, which represented low-risk zone.

In the Body Composition (BC) factor, total body fat percentage and total body water percentage items were assessed using body composition analyser. The body fat percentage of the participant was 30.5%, which was >25.1% making him fall in high-risk zone. Total body water percentage was 44.5%, which was <51.9% making him fall in high risk zone.

In the Low Risk Factor Foods (LRF) factor, intake of carbohydrates, fruits and vegetables and fibre on a daily basis were noted. The participant was consuming 393 g of carbohydrates daily, which was <440 g, depicting low risk zone. Only one serving of fruits and vegetables on daily basis by the participant put him under high-risk zone as he was consuming <3 servings. The intake of fibre was 36.1 g, which was between 26 and 50 g reading, depicting borderline risk zone.

In the High Risk Factor Foods (HRF) factor, intake of fats, sugar- added and refined and sodium on an everyday basis were noted. The participant was consuming 63.3 g fats every day, which was >50.1 g, putting him under high-risk zone. The intake of sugar was 11 tsp., which was >10tsp, again putting him under high-risk zone. The intake of sodium was 1.6 g, which was <2 g, showing low-risk zone.

In the Mental Health And Family and Personal History (MHFPH) factor, mental health and family and personal history of any disease were noted. Scoring of 24 questions in the scale under mental health item were calculated. The total score of the participant was 44, which was in between score of 37–60 depicting borderline risk zone. In family history of disease item, it was assessed that the participant's parents were suffering from two lifestyle disorders, showing borderline risk zone. In personal history of any disease item, the participant was not suffering from any disorder, depicting low risk zone.

In the Sleep and Physical Activity (SPA) factor, the participant was sleeping for seven hours in a day, showing borderline risk zone as he had sound sleep for only 6–8 h. In the physical activity item, it was assessed that the participant engaged in 75 min of physical activity daily, which was >60 min, depicting low risk zone.

Thus, in totality, the 17-year-old male participant had six items falling under low-risk zone, five items falling under borderline risk zone and five items falling under high-risk zone as seen in Table 4. Identification of borderline and high risk zone factors is pertinent so that those factors can be addressed and prevented by appropriate intervention and treatment in an early phase. Transformation to a healthy lifestyle pattern is the key to good health.

**Table 4:** Sample scoring sheet of 17 year old male adolescent.

Factor	Items	Scoring	
SOCIO- ECONOMIC CRITERIA (SEC)	Education of Head	4	Upper Middle Income Group
	Occupation of Head	4	
	Family Income (per month)	2	
Total		10	
**FACTOR**	**ITEMS**	**SCORING**	**RISK ZONE**
NUTRITIONAL STATUS (NS)	BMI	18.9 kg/m^2^	Borderline
	Waist Circumference	82 cm/ 0.92 m	Low
	Blood Pressure	118/78 mmHg	Low
BODY COMPOSITION (BC)	Body fat %	30.5%	High
	Total Body Water %	44.5%	High
LOW RISK FACTOR FOODS (LRF)	Carbohydrates	393 g	Low
	Fruits and Vegetables	1 serving	High
	Fibre	36.1 g	Borderline
HIGH RISK FACTOR FOODS (HRF)	Fats	63.3 g	High
	Sugar- added and refined	11 tsp	High
	Sodium	1.6 g	Low
MENTAL HEALTH AND PERSONAL AND FAMILY HISTORY (MHFPH)	Mental Health	44	Borderline
	Family History of any Disease	2	Borderline
	Personal History of any Disease	0	Low
SLEEP AND PHYSICAL ACTIVITY (SPA)	Sleep	7 h	Borderline
	Physical Activity	75 min per day	Low

Know your number of risk factors.

Low risk- 6 (waist circumference, Blood Pressure, carbohydrates intake, sodium intake, personal history of any disease and physical activity).

Borderline risk- 5 (BMI, fibre intake, mental health, family history of any disease and sleep).

High risk- 5 (body fat %, total body water %, fruits and vegetables intake, fats intake and sugar intake).

### 3.5 Criterion validity

The developed scale to assess risk factors of lifestyle diseases was correlated with a previously validated scale- Indian Adolescent Health Questionnaire by Long et al. [27] on 10% of the same participants as seen in Table 5 and Fig. 5.

**Table 5:** Correlation analyses between the standardized scale.

VARIABLES	Physical Health	Physical Activity	Nutrition	Hygiene	Medical care and medical history	HIV	Violence, abuse and injury	Family and home environment	School environment	cigarette, tobacco, alcohol and drugs	Strengths and difficulties	Nutritional status	Low risk factor foods	Body composition	High risk factor foods	Mental health and family and personal history of any disease	Sleep and physical activity
Physical Activity	−0.43***	..	..	..	..	..	..	..	..	..	..	..	..	..	..	..	..
Nutrition	−0.234	0.483***	..	..	..	..	..	..	..	..	..	..	..	..	..	..	..
Hygiene	0.217	0.146	−0.09	..	..	..	..	..	..	..	..	..	..	..	..	..	..
Medical care and medical history	−0.322**	0.359**	0.732***	−0.497***	..	..	..	..	..	..	..	..	..	..	..	..	..
HIV	0.217	0.146	−0.09	1***	−0.497***	..	..	..	..	..	..	..	..	..	..	..	..
Violence, abuse and injury	−0.459***	0.347**	0.596***	−0.136	0.631***	−0.136	..	..	..	..	..	..	..	..	..	..	..
Family and home environment	−0.282*	0.327**	0.614***	−0.066	0.634***	−0.066	0.361**	..	..	..	..	..	..	..	..	..	..
School environment	−0.256*	−0.106	−0.026	−0.128	−0.316**	−0.128	0.236*	−0.286*	..	..	..	..	..	..	..	..	..
cigarette, tobacco, alcohol and drugs	0.147	−0.221	−0.38**	0.276*	−0.431***	0.276*	−0.365**	−0.513***	−0.019	..	..	..	..	..	..	..	..
Strengths and difficulties	−0.14	−0.071	0.001	−0.266*	−0.058	−0.266*	0.146	−0.138	0.508***	−0.024	..	..	..	..	..	..	..
Nutritional status	0.41***	−0.245*	−0.064	0.105	−0.171	0.105	−0.066	−0.05	0.058	−0.081	0.036	..	..	..	..	..	..
Low risk factor foods	−0.405***	0.307**	0.22	0.175	−0.068	0.175	0.245*	−0.082	0.397***	−0.023	0.24	−0.124	..	..	..	..	..
Body composition	0.159	0.025	−0.313**	0.108	−0.026	0.108	−0.172	0.025	−0.491***	0.003	−0.355**	−0.049	−0.219	..	..	..	..
High risk factor foods	−0.172	0.262*	0.223	0.098	0.248*	0.098	0.493***	0.006	−0.021	0.027	−0.324**	−0.019	0.223	0.165	..	..	..
Mental health and family and personal history of any disease	0.242	−0.283*	−0.269*	0.126	−0.065	0.126	−0.415***	−0.004	−0.453***	0.335**	−0.2	0.066	−0.327**	0.269*	−0.314**	..	..
Sleep and physical activity	0.034	0.102	0.279*	−0.026	0.344**	−0.026	0.143	0.245*	−0.29*	−0.26*	0.024	0.234	−0.042	−0.048	−0.026	0.245*	..
Socio-economic criteria	−0.306**	0.178	−0.01	0.215	−0.15	0.215	0.016	−0.08	0.062	0.036	−0.077	0.105	0.313**	−0.117	0.05	−0.174	−0.082

**p < 0.01, *p < 0.

### 3.6 Discussion of correlation analysis between the standardized scale

The current scale was correlated against 12 modules of Indian Adolescent Health Questionnaire (IAHQ) by Long et al. [27] These modules were demography, physical health, physical activity, nutrition, hygiene, medical care, tobacco, alcohol and drugs, domestic violence and violence, injury and mental health, family, home and school environment. This tool was by far the closest tool to measure criterion validity as per review of literature that measured Indian Adolescents Health in line with RFLDS. The whole purpose of developing the tool was to encompass majority of the risk factors in a single tool to assess risk factors of lifestyle disorders.

Table 5 shows validation of current scale with IAHQ. Statistical significance was observed between physical health parameter of IAHQ with Nutritional Status (NS) (*r* = 0.41,*p* < 0.01**), Low Risk Factor (LRF) Foods (*r* = −0.405,*p* < 0.01**) and Socio Economic Criteria (SEC) factors (*r* = −0.306,*p* < 0.01**). In the physical activity parameter of IAHQ, negative correlation with Nutritional Status (NS) (*r* = −0.245,*p* < 0.05*) was seen depicting higher the levels of physical activity, lower the Body Mass Index, waist circumference and blood pressure. Negative correlation with Mental Health and Family and Personal History (MHFPH) factor of the current tool (*r* = −0.283,*p* < 0.05*) meant that higher the physical activity levels, lesser the probability of having history of lifestyle disorders and lower would be the score of mental health parameter depicting better mental health. The physical activity parameter was also observed to be statistically highly significant with Low Risk Factor Foods (LRF) (*r* = 0.307,*p* < 0.01**) and statistically significant with High Risk Factor Foods (HRF) (0.262,*p* < 0.05*). With the nutrition parameter of IAHQ, Body Composition (BC) (*r* = −0.313,*p* < 0.01*) and Mental Health and Family and Personal History (MHFPH) factor (*r* = −0.269, *p* < 0.05*) had shown statistically significant negative correlation. This indicated that lower the nourishment, higher the body composition values, i.e. higher the body fat percentage levels, lower the total body water percentage levels in the body. Also, lower the nutritional status, greater the probability of mental health scores depicting mental ill health as well as history of lifestyle disorders. The nutrition parameter of IAHQ had been observed to be statistically significantly correlated to Sleep and Physical Activity (SPA) factor of the current scale (*r* = 0.279,*p* < 0.05*). No statistical significance was observed between nutrition parameter of IAHQ with the nutritional factors of the current scale. This might be because in the IAHQ, the questions under the nutrition parameter were related to foods that maybe eaten, and eating and drinking habits whereas Low Risk Factor Foods (LRF) and High Risk Factor Foods (LRF) factors referred to the total intake of food components.

Overall, it was observed that the newly developed scale was in check with a previous scale and at the same time was not similar. Further, the current scale focused on assessing risk factors of lifestyle disorders. Parameters such as hygiene, family environment and school environment, HIV of IAHQ were not incorporated in the current construct because they did not fall in the purview of the present measure. However, extent of the study kept broadening with passage of time and these factors might be researched upon with lifestyle disorders in the future.

The developed validated scale was named as Risk Factors Lifestyle Disorders Scale (RFLDS). The participants taken in the present research fell in the age group of 16–18 years from the Tricity- Chandigarh, Panchkula and Mohali, thereby limiting its generalizability.

In fact, there still is need to explore the relevance of RFLDS in other regions, in different regional languages and age groups. An attempt has been made to identify and incorporate all the risk factors paving way towards lifestyle disorders in the most concise way to know and maintain a healthy lifestyle.

## 4 Limitations and future implications

The study assessed participants in the 16–18 years age bracket limiting its generalizability. There also maybe instances of social desirability among participants showing that they are following correct practices. The criterion validation had been conducted on 10% same participants and should thus be considered preliminary. Further, to evaluate generalizability, complete criterion validity shall be repeated and confirmatory factor analysis be conducted in future studies. Despite these limitations, the scale shows ample proof of it being valid, especially in the current scenario. The study recommends researchers to use Risk Factors Lifestyle Disorders Scale (RFLDS) and measure the risk factors of Non-Communicable Diseases as low, borderline and high risk zones for criterion validity. There are several future implications of this study wherein holistic health might be understood under the umbrella of lifestyle disorders. Keeping in mind, the Sustainable Development Goal 3.4, the study provides a potentiality for quick assessment, addressal of various risk factors of lifestyle disorders into low, borderline and high risk zones. After identification, the requisite risk factors might be prevented at the earliest by appropriate treatment and lifestyle changes. It is still necessary to continuously refine and re-test RFLDS to determine reliability coefficients. Multi-region school validation including rural district might be done. Translation as well as cognitive interviewing with 5 to 10 students per language might also be carried out. The current comprehensive scale would help to improve welfare of societies across various cultural realms. Further, RFLDS might be used as a screening tool in clinics, public health care systems, educational institutions as well as by researchers to recognize risk factors of lifestyle diseases worldwide.

## 5 Conclusion

The major focus while constructing the RFLDS was that all the relevant items affecting lifestyle disorders were put together under one umbrella. It was a challenge to put down all the risk factors together in line with each other as they were diverse. Composite scoring was done to bring about uniformity in every item by converging them to low, borderline and high-risk zones of lifestyle disorders, be it BMI, physical activity or mental health. The scale was made, keeping in mind the vast studies on lifestyle diseases as well as the shortcomings of the previously established scales.

The Risk Factors Lifestyle Disorders Scale- RFLDS is an attempt to develop a comprehensive scale to identify the risk factors of various lifestyle disorders to know whether the individual falls under low, borderline or high-risk zone with respect to each item. The scale is self-sufficient, as the participant after filling in the information directly, will know his or her risk factors. It is envisaged that RFLDS- Risk Factors Lifestyle Disorders Scale would be used as a screening tool for risk assessment and prevention of lifestyle disorders among adolescents as well as other age groups. This would also assist future researches by analysing the burden quickly and effectively. Because of immediate assessment through RFLDS, preventive measures could be adopted quickly, which would help in reduction of the mounting pressures on healthcare systems.

## Author contributor

Data collection was undertaken by KS. The data has been accessed and verified by KS and RP. KS and RP has the access to the data that underwent statistical analysis and led to generation of tables and figures. KS and RP have drafted the manuscript for final submission.

## CRediT authorship contribution statement

**Kalyani Singh:** Writing – review & editing, Writing – original draft, Visualization, Validation, Software, Resources, Project administration, Methodology, Investigation, Formal analysis, Data curation, Conceptualization. **Ritu Pradhan:** Writing – review & editing, Writing – original draft, Visualization, Validation, Supervision, Software, Resources, Project administration, Methodology, Investigation, Formal analysis, Data curation, Conceptualization.

## Funding

None.

## Declaration of competing interest

The authors have no declared conflicts of interest.

## Appendix A Supplementary data

Supplementary material 1: RFLDS- Brief User Guide.

Supplementary material 2: RFLDS- Scoring Sheet.

Supplementary material 3: RFLDS- Participant sample sheet of 17 year old male adolescent.

Supplementary material 4: Risk Factors Lifestyle Disorders Scale (RFLDS).
