# Supplementary material for: Development and validation of risk factors lifestyle disorders scale (RFLDS): A cohort study
Source: Dialogues Health. 2026 May 3;8:100307. doi: 10.1016/j.dialog.2026.100307 (PMC13186033; doi:10.1016/j.dialog.2026.100307)
Supplement: Supplementary file 1 — Supplementary material 1: RFLDS- Brief User Guide. [file mmc1.pdf]

RISK FACTORS LIFESTYLE DISORDERS SCALE (RFLDS)

BRIEF USER GUIDE

The current scale aims for quick and easy identification of risk factors of lifestyle disorders for addressal, prevention and screening. It has been developed to educate and improve the health status as well as foster quality of life. The risk factors of lifestyle disorders have been bifurcated into low, borderline and high risk zones. After identification, the requisite risk factors might be prevented at the earliest by appropriate treatment and lifestyle changes.

WHO SHOULD ADMINISTER?

- RFLDS can be self-administered by the individual to identify which risk factors of his/her fall in low, borderline and high risk zones. The scale can also be administered by an nutritionists, health care professionals/ workers, academicians, doctors, clinicians, nurses etc. as well.

ESTIMATED TIME

- 10-12 minutes

MINIMAL EQUIPMENT

- Pen and paper
- Height rod
- Calculator (BMI calculation)
- Body composition analyser
- Non-stretchable measuring tape
- Blood pressure apparatus
- Additional paper for 24 hour dietary recall

ACTIONS TRIGGERED BY HIGH RISK ZONES

- Constant assessment and apt interventions are necessary to prevent lifestyle disorders amongst them to maintain health status. Identification of high risk zone factors is pertinent so that those factors can be addressed and prevented by appropriate intervention and treatment in an early phase. Transformation to a healthy lifestyle pattern is the key to good health.
