# Supplementary material for: Development and validation of risk factors lifestyle disorders scale (RFLDS): A cohort study
Source: Dialogues Health. 2026 May 3;8:100307. doi: 10.1016/j.dialog.2026.100307 (PMC13186033; doi:10.1016/j.dialog.2026.100307)
Supplement: Supplementary file 2 — Supplementary material 2: RFLDS- Scoring Sheet. [file mmc2.pdf]

SCORING SHEET- RISK FACTORS LIFESTYLE DISORDERS SCALE (RFLDS)

**FACTOR**	**ITEMS**	**SCORING**	
**SOCIO- ECONOMIC CRITERIA (SEC)**	EDUCATION OF HEAD<br>OCCUPATION OF HEAD<br>FAMILY INCOME	1-4	Lower Income Group
		5-8	Lower Middle Income Group
		9-13	Upper Middle Income Group
		14-17	Upper Income Group
**FACTOR**	**ITEMS**	**SCORING**	**RISK ZONE**
**NUTRITIONAL STATUS (NS)**	BMI (kg/m^2)	<18.5kg/m^2	Low
		18.6-22.9 kg/m^2	Borderline
		>23.0 kg/m^2	High
	Waist Circumference (in mts)	Male- <0.95	Low
		Female- <0.80	
		Male- 0.96-1.01	Borderline
		Female- 0.81-0.87	
		Male- >1.02	High
		Female- >0.88	
	Blood Pressure	Systolic- <120mm Hg	Low
		Diastolic- <80 mm Hg	
		Systolic- 121-129mm Hg	Borderline
		Diastolic- 81-89mm Hg	
		Systolic- >130 mm Hg	High
		Diastolic- >90 mm Hg	
**BODY COMPOSITION (BC)**	Body Fat %	Male- <20.9%	Low
		Female- <30.9%	
		Male- 21-25%	Borderline
		Female- 31-33%	
		Male- >25.1%	High
		Female- >33.1%	
	Total Body Water %	Male- >66.1%	Low
		Female- >63.1%	
		Male- 52-66%	Borderline
		Female- 49-63%	
		Male- <51.9%	High

		Female- <48.9%	
**LOW RISK FACTOR FOODS (LRF)**	Carbohydrates (gm)	Male- <440gm	Low
		Female- <320gm	
		Male- 440-460gm	Borderline
		Female- 320-340gm	
		Male- >460gm	High
		Female- >340gm	
	Fruits and Vegetables (100 gm= 1 serving)	>5	Low
		4	Borderline
		<3	High
	Fibre (gm)	Male- >51gm	Low
		Female- >39gm	
		Male- 26-50gm	Borderline
		Female- 26-38gm	
		Male- <25gm	High
		Female- <25gm	
**HIGH RISK FACTOR FOODS (HRF)**	Fats (gm)	Male- <44.9gm	Low
		Female- <29.9 gm	
		Male- 45-55gm	Borderline
		Female- 30-40gm	
		Male- >55.1gm	High
		Female- >40.1gm	
	Sugar – added and refined 1tsp= 5gm	<5tsp	Low
		6-9 tsp	Borderline
		>10 tsp	High
	Sodium (gm)	<2gm	Low
		2gm	Borderline
		<2gm	High
**MENTAL HEALTH AND PERSONAL AND FAMILY HISTORY (MHFPH)**	Mental Health	<36	Low
		37-60	Borderline
		>61	High
	Family History of any Disease	0	Low
		1-3	Borderline
		>3	High
	Personal History of any Disease	0	Low
		1-3	Borderline
		>3	High

**SLEEP AND PHYSICAL ACTIVITY (SPA)**	Sleep (hours)	>8 hours	Low
		6-8 hours	Borderline
		<6 hours	High
	Physical Activity (minutes per day)	>60 min	Low
		60 min	Borderline
		<60 min	High
