# Supplementary material for: Development and validation of risk factors lifestyle disorders scale (RFLDS): A cohort study
Source: Dialogues Health. 2026 May 3;8:100307. doi: 10.1016/j.dialog.2026.100307 (PMC13186033; doi:10.1016/j.dialog.2026.100307)
Supplement: Supplementary file 4 — Supplementary material 4: Risk Factors Lifestyle Disorders Scale (RFLDS). [file mmc4.pdf]

RISK FACTORS LIFESTYLE DISORDERS SCALE (RFLDS)

Ms. Kalyani Singh[1] and Dr. Ritu Pradhan[2]

1- **Research Scholar, Panjab University, Chandigarh, India**
2- **Associate Professor, Government Home Science College, Chandigarh, India**

<u>Instructions</u>

The current scale assesses the risk factors of chronic lifestyle disorders. Kindly fill the scale to assess risk factors for chronic lifestyle disorders. The scale will categorize the risk factors as low, borderline or high-risk zone.

Wishing you all happy health!!

SOCIO-ECONOMIC CRITERIA (SEC)

**EDUCATION OF HEAD (Circle it)**	Illiterate<br>1	Primary<br>2	Middle<br>3	10th<br>4	12th and Diploma<br>5	Graduate<br>6	Post Graduate/ Diploma and above<br>7
**OCCUPATION OF HEAD (Circle it)**	Unemployed<br>1	Unskilled- Loader, Un-loader, Puda Maker and Chowkidar<br>2	Semi skilled- sorter/ checker, bhattiwala, driver (light vehicle), typist, billman, clerk<br>3	Skilled- driver (heavy worker), accountant, munim, cashier, store keeper, head clerk, godown keeper<br>4	Professional<br>5		
**FAMILY INCOME (per month) (Circle it)**	<25,000/- per month<br>1	25,001-50,000/- per month<br>2	50,001-1,00,000/- per month<br>3	1,00,001-1,50,000/- per month<br>4	>1,50,001/- per month<br>5		

Add the numbers of circled item

Socio Economic Criteria (SEC)	SCORE
Lower Income Group	1-4
Lower Middle Income Group	5-8
Upper Middle Income Group	9-13
Upper Income Group	14-17

1. NUTRITIONAL STATUS (NS)

		Reading			L/B/H
		**1(L)**	**2(B)**	**3(H)**	
**BMI (Kg/m^2)**	_______kg/m^2	<18.5kg/m^2	18.6-22.9 kg/m^2	>23.0 kg/m^2	
**WAIST CIRCUMFERENCE (in mts)**	M	<0.95	0.96-1.01	>1.02	
	F	<0.80	0.81-0.87	>0.88	
**BLOOD PRESSURE SYSTOLIC**	_____mm Hg	<120mm Hg	121-129mm Hg	>130 mm Hg	
**DIASTOLIC**		<80 mm Hg	81-89mm Hg	>90 mm Hg	

2. BODY COMPOSITION (BC)

		Reading			L/B/H
		**1(L)**	**2(B)**	**3(H)**	
**Body Fat %**	M	<20.9%	21-25%	>25.1%	
	F	<30.9%	31-33%	>33.1%	
**Total Body Water %**	M	>66.1%	52-66%	<51.9%	
	F	>63.1%	49-63%	<48.9%	

3. LOW RISK FACTOR FOODS (LRF)

S.No	Nutrients			Reading			L/B/H
				1(L)	2(B)	3(H)	
1	**Carbohydrates**		M	<440gm	440-460gm	>460gm	
			F	<320gm	320-340gm	>340gm	
2	**Fruits and Vegetables**	Serving (100gm) __________		>5	4	<3	
3	**Fibre**		M	>51gm	26-50gm	<25gm	
			F	>39gm	26-38gm	<25gm	

4. HIGH RISK FACTOR FOODS (HRF)

S.No	Nutrients			Reading			L/B/H
				1(L)	2(B)	3(H)	
1	**Fats**	_______(total)	M	<44.9gm	45-55gm	>55.1gm	
			F	<29.9 gm	30-40gm	>40.1gm	
2	**Sugar – added and refined**	_____________		<5tsp	6-9 tsp	>10 tsp	
3	**Sodium**	_____________		<2gm	2gm	>2gm	

Note- 1 tsp= 5gm

5. MENTAL HEALTH AND FAMILY AND PERSONAL HISTORY (MHFPH)

MENTAL HEALTH

S.No.		1 (never)	2 (sometimes)	3 (always)	L/B/H
1.	Little things upset me				
2.	I feel useless when I am not able to achieve what I want.				
3.	I have a tendency to make a big deal of circumstances				
4.	I feel breathless even without doing exercise				
5.	Its tough for me to stay calm				
6.	I sweat quite a lot even when it is not hot				
7.	I let down myself when I don't score well				
8.	My parents push me to study hard.				
9.	I feel my results map my future				
10.	I let down my teacher when I don't score well				
11.	I let down my parents when I don't score well				
12.	I have sleepless nights due to tension of achieving my goals.				
13.	I feel dizzy				
14.	I worry without any reason				
15.	I get upset in no time				
16.	My parents feel I am not good in studies				
17.	I get panicky very quickly				
18.	I feel I am moody				
19.	I feel I am short tempered				
20.	My parents have high expectations from me.				
21.	I stay nervous/ edgy				
22.	I have felt high pulse rate even when I am not doing any workout				
23.	My parents are never satisfied with my academic performance.				
24.	I feel low on patience				
	TOTAL SCORE				
		**<36 (L)**	**37-60 (B)**	**>61 (H)**	

HISTORY OF ANY DISEASE (Tick mark)

Does any of your parent/ parents suffer from any of the following disease/diseases

	**Disease**	**YES**		**NO**	**L/B/H**
1	Type 1 diabetes				
2	Type 2 diabetes				
3	Heart disease __________				
4	Hypertension				
5	Overweight				
6	Obese				
		**0 (L)**	**1-3 (B)**	**>3 (H)**	

Do you suffer from any of the following disease/diseases

	**Disease**	**YES**		**NO**	**L/B/H**
1	Type 1 diabetes				
2	Type 2 diabetes				
3	Heart disease __________				
4	Hypertension				
5	Overweight				
6	Obese				
		**0 (L)**	**1-3 (B)**	**>3 (H)**	

6. SLEEP AND PHYSICAL ACTIVITY (SPA)

SOUND SLEEP

S. No.			**Per Day**			**L/B/H**
			**1(L)**	**2(B)**	**3(H)**	
1.	No. of total sleep hours	_____ hours	>8 hrs	6-8 hrs	<6 hrs	

PHYSICAL ACTIVITY INFORMATION

S. No.			**Mins Per Day**		
1.	Do you exercise?	Yes/ No			
2.	Do you do any vigorous activities like aerobics, heavy lifting or high-speed bicycling?	Yes/ No			
3.	Do you do any moderate activities like normal speed bicycling, playing games (cricket, tennis, basket ball, kabaddi, etc) carrying light weights?	Yes/ No			
4.	Do you do any light activities like walking (brisk walk, going to the market, religious place, walking and talking on phone etc)	Yes/ No			
		**1 (L)**	**2 (B)**	**3 (H)**	**L/B/H**
	Average Minutes per day	>60 min	60 min	<60min	

KNOW YOUR NUMBER OF RISK FACTORS

Low -

Borderline-

High -
